# A Diallel of the Mouse Collaborative Cross Founders Reveals Strong Strain-Specific Maternal Effects on Litter Size

**DOI:** 10.1534/g3.118.200847

**Published:** 2019-03-15

**Authors:** John R. Shorter, Paul L. Maurizio, Timothy A. Bell, Ginger D. Shaw, Darla R. Miller, Terry J. Gooch, Jason S. Spence, Leonard McMillan, William Valdar, Fernando Pardo-Manuel de Villena

**Affiliations:** * Department of Genetics, University of North Carolina, Chapel Hill, North Carolina, 27599-7265; † Curriculum in Bioinformatics and Computational Biology, University of North Carolina, Chapel Hill, North Carolina, 27599-7265; ‡ Lineberger Comprehensive Cancer Center, University of North Carolina, Chapel Hill, North Carolina, 27599-7265; § Department of Computer Science, University of North Carolina, Chapel Hill, North Carolina, 27599-7265

**Keywords:** F1 cross, additive heritability, fertility, BayesDiallel, zero-truncated Poisson, variance projection, Multiparent Advanced Generation Inter-Cross (MAGIC), multiparental populations, MPP

## Abstract

Reproductive success in the eight founder strains of the Collaborative Cross (CC) was measured using a diallel-mating scheme. Over a 48-month period we generated 4,448 litters, and provided 24,782 weaned pups for use in 16 different published experiments. We identified factors that affect the average litter size in a cross by estimating the overall contribution of parent-of-origin, heterosis, inbred, and epistatic effects using a Bayesian zero-truncated overdispersed Poisson mixed model. The phenotypic variance of litter size has a substantial contribution (82%) from unexplained and environmental sources, but no detectable effect of seasonality. Most of the explained variance was due to additive effects (9.2%) and parental sex (maternal *vs.* paternal strain; 5.8%), with epistasis accounting for 3.4%. Within the parental effects, the effect of the dam’s strain explained more than the sire’s strain (13.2% *vs.* 1.8%), and the dam’s strain effects account for 74.2% of total variation explained. Dams from strains C57BL/6J and NOD/ShiLtJ increased the expected litter size by a mean of 1.66 and 1.79 pups, whereas dams from strains WSB/EiJ, PWK/PhJ, and CAST/EiJ reduced expected litter size by a mean of 1.51, 0.81, and 0.90 pups. Finally, there was no strong evidence for strain-specific effects on sex ratio distortion. Overall, these results demonstrate that strains vary substantially in their reproductive ability depending on their genetic background, and that litter size is largely determined by dam’s strain rather than sire’s strain effects, as expected. This analysis adds to our understanding of factors that influence litter size in mammals, and also helps to explain breeding successes and failures in the extinct lines and surviving CC strains.

A critical part of fitness is optimizing the balance between numbers of offspring, offspring body size, and reproductive timing, leading to strong selection for these traits (Smith and Fretwell 1974; Beauchamp and Kacelnik 1990). Fundamental fitness traits, like litter size (*i.e.*, the number of pups born per litter), should have low contributions of genetic variance due to rapid fixation of alleles that increase fitness (Mousseau and Roff 1987; Price and Schluter 1991; Merilä and Sheldon 1999; Stirling *et al.* 2002; Peripato *et al.* 2004). Estimates of litter size heritability vary from 5 to 20% in mouse, rabbit, and porcine populations (Falconer 1960; Avalos and Smith 1987; Falconer and Mackay 1996; Johnson *et al.* 1999; García and Baselga 2002; Peripato *et al.* 2004; Gutiérrez *et al.* 2006) leading to the majority of variation in litter size being non-heritable. Non-heritable factors include seasonality, food availability, paternal and maternal effects, dominance and epistasis, as well as unknown factors. Additionally, sexual conflict over parental investment may exist between females and males, and this in turn could affect litter size (Trivers 1974; Hager and Johnstone 2003). These factors make it difficult to obtain clear measurements of litter size in wild populations. A controlled laboratory environment is therefore necessary to measure genetic effects on litter size (Jinks and Broadhurst 1963; Roberts 1960; Bandy and Eisen 1984; Hoornbeek 1968; Peripato *et al.* 2004; Gutiérrez *et al.* 2006; Varona and Sorensen 2014).

The Collaborative Cross (CC) and its eight founder strains are an important resource for studying complex traits, for establishing mouse models for human disease, and for understanding the mouse Diversity Outbred (DO), which originated from the CC (Ferris *et al.* 2013; Chesler 2014; Rogala *et al.* 2014; Rasmussen *et al.* 2014; Gralinski *et al.* 2015, 2017; Schoenrock *et al.* 2017; Maurizio *et al.* 2018). The CC and its founder strains are also useful for studying reproductive ability due to their established record of breeding successes and failures. For example, litter size in the early CC lines shows a steady decline over the first six generations of inbreeding (Philip *et al.* 2011). In addition, nearly 95% of all CC lines have become extinct, primarily due to subspecific genomic incompatibilities (Shorter *et al.* 2017). Although standard reproductive phenotypes have been measured, mostly in male CC founders (Odet *et al.* 2015), the genetic and non-genetic control over CC breeding success has never been thoroughly characterized, and is likely to contain vital information that may be used to improve CC breeding in the future.

Given the growing popularity of the CC as a community resource, we investigated reproductive ability across the eight founders of the CC to better understand the genetic and non-genetic factors that affect their fertility and breeding. Using an 8×8 diallel design, we measured weaned litter sizes from 4,448 litters arising from 62 crosses across four years of breeding. Adapting a recently developed statistical model of diallel effects (Lenarcic *et al.* 2012), we quantified both the genetic contributions that shape litter size and the contributions of several environmental factors. Our results provide a detailed account of breeding patterns across and between the eight founders. This experiment also informs us about genetic combinations that are highly or marginally productive in the CC, helping us to better understand CC fertility problems and line extinction.

## Materials and Methods

The mouse inbred strains used in these experiments are the eight founder strains of the Collaborative Cross (CC) (Collaborative Cross Consortium 2012). The founders of the CC include five classical strains, A/J (AJ), C57BL/6J (B6), 129S1/SvImJ (129S1), NOD/ShiLtJ (NOD), NZO/HlLtJ (NZO), and three wild-derived strains, CAST/EiJ (CAST), PWK/PhJ (PWK), and WSB/EiJ (WSB). Mice originated from a colony maintained by Gary Churchill at the Jackson Laboratory, and were transferred to the FPMV lab at the University of North Carolina (UNC) in 2008. The original colony also produced most of the G1 breeders that populated the inbred funnels at ORNL, TAU and Geniad (Srivastava *et al.* 2017). All mice described here were reared by the FPMV lab at UNC. Mice were bred at the UNC Hillsborough vivarium from 2008-2010 and bred at the UNC Genetic Medicine Building (GMB) vivarium from 2010-2012. A total of 4,448 litters resulting from crosses between 1,478 individual dams and 1,238 individual sires were born from all eight inbred crosses and 54 of 56 reciprocal F1 hybrid crosses, excluding hybrids between NZO×CAST and NZO×PWK, which are known unproductive crosses (Chesler *et al.* 2008). The directions of all crosses are described as female by male (*i.e.*, dam.strain × sire.strain), unless otherwise noted. Litter size and sex were determined at weaning by visual inspection. Animals were kept on a 14-hour, 10-hour light/dark schedule with lights turned on at 7:00 am; temperature was maintained at 20°-24° with relative humidity between 40–50%. Mice were housed in standard 20×30-cm ventilated polysulfone cages with standard laboratory grade Bed-O-Cob bedding. Water and Purina Prolab RMH3000 were available *ad libitum*. Mouse chow was supplemented with Fenbendazole (Feb 2010) two weeks before and two weeks after transportation to the GMB facility to eliminate possible pinworms. Selamectin treatment was dropped onto the coats of mice before transfer to remove mites from the cages. These treated cages were not opened until after their arrival at UNC GMB. All animal rearing and breeding was conducted in strict compliance with the *Guide for the Care and Use of Laboratory Animals* (Institute of Laboratory Animals Resources, National Research Council 1996, https://www.ncbi.nlm.nih.gov/books/NBK232589/). The Institutional Animal Care and Use Committee of the University of North Carolina approved all animal use and research described here.

## Statistical Analysis

### Testing environmental interactions

Significant environmental interactions were determined using ANOVA, using JMP 12 software (JMP, Version 12. SAS Institute Inc., Cary, NC, 1989-2007). Tested effects included a season effect (average weaned litter size in each month over every year), non-seasonal factors using a year-by-month effect (average weaned litter size for all months across all years), and a litter order effect (average weaned litter size in each subsequent dam litter). We performed the ANOVA on litter size counts from the eight inbred matings only, because of their robust sample sizes throughout the four years of breeding.

### Diallel analysis of litter size

To estimate the overall contributions of heritable factors affecting litter size in our population, we adapted a previously published linear mixed model, BayesDiallel (Lenarcic *et al.* 2012), which performs this estimation for continuous phenotypes, to the setting of a discrete count-based phenotype. To do this we reimplemented the BayesDiallel Gibbs sampler as a generalized linear mixed model (GLMM) using the R software package MCMCglmm (Hadfield 2010).

Let $y_{i}$ be the number of pups born to litter *i*, where $y_{i}$ is a positive integer (zeros are not observed). For any categorical variable *x*, let the notation x[i] indicate the value of *x* relevant to *i*: in particular, let h[i] denote litter *i*’s batch h=1,…,48; let r[i] denote its parity order r=1,…,12; and let (j,k)[i] denote its parentage, defined by maternal strain *j* and paternal strain *k*, where $\left(j,k\right)\in {\left\{1,\ldots ,8\right\}}^{2}$. The effect of parental strains on $y_{i}$ was modeled using an overdispersed zero-truncated Poisson (ZTP) regression (data scales of the model in brackets):

$$\begin{array}{l} y_{i}∼\text{ZTPois}\left(\lambda_{i}\right)\;\;\;\;\;\;\;\;\;\;\;\;\;\;\;\;\;\;\;\;\;\;\;\;\;\;\;\left[\text{observed}\right]\\ \lambda_{i}={\text{g}}^{\text{-1}}\left(\ell_{i}\right)\;\;\;\;\;\;\;\;\;\;\;\;\;\;\;\;\;\;\;\;\;\;\;\;\;\;\;\;\;\;\;\;\;\left[\text{expected}\right]\\ \ell_{i}=\eta_{i}+\varepsilon_{i}\;\;\;\;\;\;\;\;\;\;\;\;\;\;\;\;\;\;\;\;\;\;\;\;\left[\text{latent}+\text{overdis}.\right]\\ \eta_{i}=\mu +{\text{order}}_{r\left[i\right]}+{\text{batch}}_{h\left[i\right]}+d^{\text{T}}{}_{\left(j,k\right)\left[i\right]}\beta \;\;\left[\text{latent}\right] \end{array}$$ (1)

where $\text{ZTPois}\left(\lambda_{i}\right)$ denotes a Poisson distribution with an expected value $E\left[y_{i}\right]=\frac{\lambda_{i}}{1-e^{-\lambda_{i}}}$ but is conditional on having observed that $y_{i}\neq 0$ (**Appendix A**), *g* is the link function g(x)=log(x), with inverse $g^{-1}\left(x\right)=e^{x}$, that relates $\lambda_{i}$ to a latent scale $\ell_{i}$, and $\eta_{i}$ is a linear predictor on that latent scale with an error term $\varepsilon_{i}∼\text{N}\left(0,\sigma^{2}\right)$ providing overdispersion. The linear predictor $\eta_{i}$ is composed of the following: an intercept *μ*; a litter (parity) order effect, modeled as the combination of a fixed effect slope, rα, and a random effect with an independent level for each litter order, *i.e.*, ${\text{order}}_{r}∼\text{N}\left(r\alpha ,\tau_{\text{order}}^{2}\right)$, where *α* is a fixed effect and $\tau_{\text{order}}^{2}$ is the variance of the random deviations around rα; a batch effect, ${\text{batch}}_{h}∼\text{N}\left(0,\tau_{\text{batch}}^{2}\right)$; and a linear predictor, $d_{\left(j,k\right)}^{\text{T}}\beta$, modeling the effect of the parental strain combination (j,k).

The contribution of parental strains, $d_{\left(j,k\right)}^{\text{T}}\beta$, is equivalent to the ‘fullu’ (full, unsexed, ‘Babmvw’) model described in (Lenarcic *et al.* 2012), namely,

$$\begin{array}{l} d_{\left(j,k\right)}^{\text{T}}\beta =\underset{\text{additive}}{\underset{︸}{a_{j}+a_{k}}}+\underset{\text{parental sex}}{\underset{︸}{m_{j}-m_{k}}}+\underset{\text{inbred penalty}}{\underset{︸}{I_{\left\{j=k\right\}}\left(\beta_{\text{inbred}}+b_{j}\right)}}\\ +\underset{\text{epistasis}}{\underset{︸}{I_{\left\{j\neq k\right\}}\left(v_{jk}+S_{\left\{j<k\right\}}\cdot w_{jk}\right)}} \end{array}$$ (2)

where subscripted variables a,m,b,v,w model the effects of specific strains or strain-pairs, $\beta_{\text{inbred}}$ models an overall effect, $I_{\left\{A\right\}}$ is an indicator and equal to 1 if *A* is true and 0 otherwise, $S_{\left\{A\right\}}$ is a sign variable equal to $\frac{1}{2}$ if *A* is true and $-\frac{1}{2}$ otherwise. In more detail, the *a* (“additive”) class represents strain-specific dosage effects, with $a_{1},\ldots ,a_{8}$ modeled as $a_{j}∼{\text{N}}_{\text{stz}}\left(0,\tau_{a}^{2}\right)$, where ${\text{N}}_{\text{stz}}$ is a normal distribution subject to the sum-to-zero constraint $\sum_{j}a_{j}=0$ [using the approach of Crowley *et al.* (2014), Appendix A]. The *m* (“parental sex”) class represents parent-of-origin effects, modeled as $m_{j}∼{\text{N}}_{\text{stz}}\left(0,\tau_{m}^{2}\right)$, where a positive value of $m_{j}$ implies that strain *j* increases litter size more when inherited through the maternal line, with the difference between maternal and paternal being $2m_{j}$ (since the design matrix entry for $m_{j}$ is coded +1 for dam and −1 for sire). The effect of being inbred is composed of an overall (fixed) effect $\beta_{\text{inbred}}$ and strain-specific (random) effects $b_{j}∼{\text{N}}_{\text{stz}}\left(0,\tau_{b}^{2}\right)$. Epistatic effects are modeled as strain-pair specific deviations, with “symmetric” effects $v_{jk}∼{\text{N}}_{\text{stz}}\left(0,\tau_{v}^{2}\right)$ representing the overall deviation from the rest of the model induced by the (unordered) strain combination *j* with *k*, and “asymmetric” effects $w_{jk}∼{\text{N}}_{\text{stz}}\left(0,\tau_{w}^{2}\right)$ modeling a further deviation induced by differences in parent-of-origin.

To provide directional parent-of-origin effects for maternal and paternal strain we defined, by reparameterization of the additive and parental sex effect, the following two additional types of effect:

$${\text{dam}}_{j}=a_{j}+m_{j}$$

$${\text{sire}}_{j}=a_{j}-m_{j}\;.$$

For example, ${\text{dam}}_{\text{B}6}=a_{\text{B}6}+m_{\text{B}6}$ is the B6-specific dam (maternal strain) effect and ${\text{sire}}_{\text{B}6}=a_{\text{B}6}-m_{\text{B}6}$ is the B6-specific sire (paternal strain) effect. Posterior samples of these quantities were obtained as a post-processing step by reparameterizing posterior samples of $a_{j}$ and $m_{j}$. Obtaining these as a post-processing step, rather than explicit modification of Equation 2, preserves the original BayesDiallel model and therefore has no effect on the effect estimates (or variance projection estimates) for the other diallel categories. (Parameter definitions summarized in Table S1.)

The decomposition of diallel effects in Equation 2 and its dam *vs.* sire reformulation have parallels in earlier diallel literature. A reduced version of the decomposition composed of additive (*a*) and symmetric epistasis (*v*) estimate, respectively, the Generalized Combining Ability (GCA) and Specific Combining Ability (SCA) described by Sprague and Tatum (1942); including also the (reciprocal) asymmetric epistasis (*w*) term recapitulates Griffing’s Method 1, Model 1 (Griffing 1956). The inbred penalty ($\beta_{\text{inbred}}$, *b*) is comparable to dominance measures defined in, for example, Hayman (1954). The parental sex effects (*m*) are akin to the maternal effects of Topham (1966) and the “extranuclear” effects in the “bio” model of Cockerham and Weir (1977) and Zhu and Weir (1996), and the bio model’s reparameterization to indirectly estimate dam and sire effects has been described in, for example, Lynch and Walsh (1998). The estimation of dam and sire effects directly, as explicit model parameters for the diallel, was proposed by Comstock and Robinson (1948, 1952). The main differences between these earlier analyses and ours are the simultaneous inclusion of all parameter groups, the use of a Bayesian random effects framework to allow all these parameters to be fitted, and the extension to modeling a non-Gaussian response.

Priors were chosen to be minimally informative. For fixed effects (*μ*, *α*, and $\beta_{\text{inbred}}$) we used $\text{N}\left(0,1\times 10^{3}\right)$. For variances of random effects ($\sigma^{2}$, $\tau_{a}^{2}$, $\tau_{m}^{2}$, $\tau_{b}^{2}$, $\tau_{v}^{2}$, $\tau_{w}^{2}$, $\tau_{\text{order}}^{2}$, $\tau_{\text{batch}}^{2}$) we used an inverse gamma distribution with scale and shape both equal to 0.001. Posterior effect estimates are presented as posterior mean (and median), and the 95% highest posterior density (HPD) interval.

In order to stably estimate the contribution of each variance class to the overall phenotype, we used the diallel variance projection [VarP; Crowley *et al.* (2014)]. This is a heritability-like measure that partitions the overall phenotypic variance of an idealized future diallel experiment into additive, parent-of-origin (parental sex), inbred (dominance), epistatic, and other random/fixed effects categories in the diallel. Rather than being based on estimated variance parameters (*e.g.*, $\tau_{a}^{2},\tau_{b}^{2},\ldots$), which are typically ill-informed by the data and thus both uncertain and sensitive to priors, the VarP uses the estimated effects themselves, both fixed (*e.g.*, $\beta_{\text{inbred}}$) and random (*i.e.*, the best linear unbiased predictors, or BLUPs, such as $a_{1},a_{2},\ldots$), since these are well-informed, precise and regularized by shrinkage. The VarP calculation involves ratios of sums-of-squares in similar fashion to $R^{2}$ but for an idealized diallel that is both complete and balanced. As an $R^{2}$-like measure, the VarP is reportable for both fixed and random effects, and includes confidence intervals arising from posterior uncertainty in those effects’ values.

VarPs were calculated both from the ZTP model described above, and, for comparison, from the standard Gaussian-outcome BayesDiallel model (using the BayesDiallel software) (**Appendix A**). The standard BayesDiallel model was applied to our data after litter size was subject to a variance-stabilizing transformation, the square root, this corresponding to the linear mixed model approximation to the ZTP,

$$\text{sqrt}\left(y_{i}\right)∼\mu +{\text{order}}_{r\left[i\right]}+{\text{batch}}_{h\left[i\right]}+d_{\left(j,k\right)\left[i\right]}^{\text{T}}\beta +\varepsilon_{i}\;.$$ (3)

### Diallel analysis of sex ratio

To model diallel effects on sex ratio we recast the BayesDiallel model as a binomial GLMM. Letting $y_{i}$ be the number of males out of a total of $n_{i}$ pups for litter *i*, we model

$$\begin{array}{l} y_{i}\;∼\text{ Binom }\left(n_{i},\;\pi_{i}\right)\;\;\;\;\left[\text{observed}\right]\\ \pi_{i}={\text{g}}^{\text{-1}}\text{(}\ell_{i}\text{)}\;\;\;\;\;\;\;\;\;\;\;\;\;\;\;\;\left[\text{expected}\right] \end{array}$$ (4)

where $\pi_{i}$ is the expected proportion of males predicted for litter *i*, the link function is g(x)=logit(x)=log(x)/(1−x), with inverse link $g^{-1}\left(x\right)={\text{logit}}^{-1}\left(x\right)=\text{expit}\left(x\right)=e^{x}/\left(1+e^{x}\right)$, and $\ell_{i}$, which represents $\pi_{i}$ on the latent scale, is modeled using the BayesDiallel hierarchy as in Equation 1.

Additional details about our statistical modeling approaches are provided in **Appendix A** (litter size) and **Appendix B** (sex ratio).

### Data Availability

File S1 contains all breeding data used for the analysis in this study. File S2 contains the scripts and software used for the analysis in this study. File S3 contains the Supplemental Figures and Tables. The data and code used to analyze the data are available at https://github.com/mauriziopaul/litterDiallel, with a static version available at https://doi.org/10.5281/zenodo.2580307. Phenotype data from this study is available on the Mouse Phenome Database (Bogue *et al.* 2018) at https://phenome.jax.org/projects/Shorter3 with persistent identifier RRID:SCR003212, Accession number MPD:623, project Shorter3. Supplemental material available at Figshare: https://figshare.com/s/fefa6216bf29b2baeb65.

## Results

### Litter size is affected by housing facility but not season

**Figure 1:**
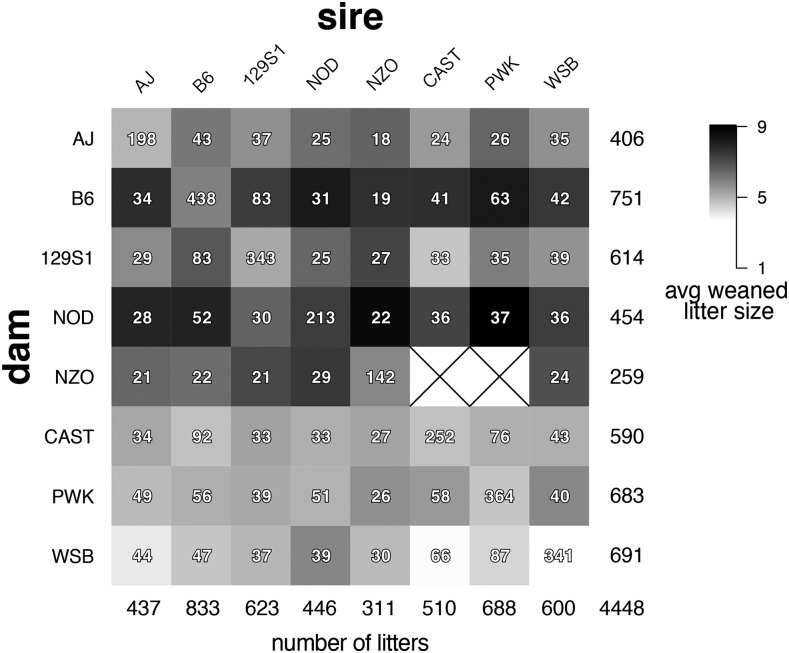
Diallel crossing scheme and weaned pup distribution. The number of litters observed per cross is given by the integers, with the largest sample sizes, along the diagonal, corresponding to the production of inbred parental strains. Column and row sums are given along the bottom row and rightmost column, respectively. A total of 4,448 litters were evaluated for this analysis, resulting in a total of 24,782 weaned pups. The shading within each box corresponds to the average number of weaned pups per litter in each cross, with averages ranging from 3.6 to 9.1 pups per litter. Litters for which no pups survived until weaning were not included in our analysis. The symbol “×” is used to indicate incompatible crosses that do not produce any litters.

We bred litters in an 8×8 inbred diallel of the CC founder strains, and generated all eight inbreds and 54 of 56 possible reciprocal F1 hybrids (Figure 1, Figure S1–S3). The eight inbred strains, displayed across the diagonal, were mated at higher frequencies both for maintenance of inbred strains and propagation of the diallel. For all 62 genetic inbred and hybrid crosses, we recorded the following information: mated pairs, wean dates, litter size at weaning, including total and sex-specific counts (File S1). This diallel cross was originally designed and maintained for the generation of F1 mice for several experimental projects (Koturbash *et al.* 2011; Aylor *et al.* 2011; Mathes *et al.* 2011; Kelada *et al.* 2012; Didion *et al.* 2012; Collaborative Cross Consortium 2012; Calaway *et al.* 2013; Crowley *et al.* 2014; Phillippi *et al.* 2014; Odet *et al.* 2015; Crowley *et al.* 2015; Morgan *et al.* 2016; Percival *et al.* 2016; Shorter *et al.* 2017; Oreper *et al.* 2017; Maurizio *et al.* 2018). As a result, certain reproductive measurements such as time between litters and maximum number of offspring per cross were necessarily biased by experimental breeding requirements. Average weaned litter size, however, is a reproductive trait that should be well-estimated independently of these factors.

We measured litter size and report the mean number of weaned pups per litter for the 62 viable crosses in the diallel (Figure 1). A wide distribution of litter sizes was observed, ranging from an average of 3.6 weaned pups for WSB×WSB crosses, to an average of 9.1 weaned pups for NOD×PWK crosses, with an overall mean of 5.5 weaned pups per litter. Examining average litter sizes of the inbred strains across all 4 years, we found neglible evidence of consistent seasonal effects ($F_{11,2283}=1.272$ *P* = 0.23) (Figure S4) but positive evidence of non-seasonal patterns: a modeled ‘year.month’ covariate significantly affected litter size ($F_{47,2247}=2.44$ *P* < 0.0001; see also Figure S5). The non-seasonal effect could be driven by the relocation of mice between vivariums, which occurred in February 2010. The housing facility may have an impact on litter size as well. To test this, we measured average litter size differences between the two housing facilities and found that two founder strains, 129S1 and WSB, had significantly larger average litter sizes at the Hillsborough facility than at UNC GMB with a difference of 5.87 to 4.42 weaned pups per litter (*P* < 0.0001) for 129S1, and 3.84 to 3.42 weaned pups per litter (*P* = 0.029) for WSB. The six other founder strains did not significantly differ in their average litter sizes between the two facilities, but tended to have smaller weaned litters at UNC GMB (see Discussion). For average litter size across the diallel, we tested for an effect of litter number (birth parity number, for a given mating pair) on the number of pups weaned in each litter. Previous research suggests that the first litter can be significantly smaller than subsequent litters due to various biological factors (De la Fuente and San Primitivo 1985). This effect was consistent in the diallel: we observed that there was significant reduction in litter size in the first litter (*P* < 0.0001) compared to the overall linear effect that parity has on reducing litter size (Figure S5).

### Litter size is moderately heritable, and maternal effects account for the majority of explained variation

**Figure 2:**
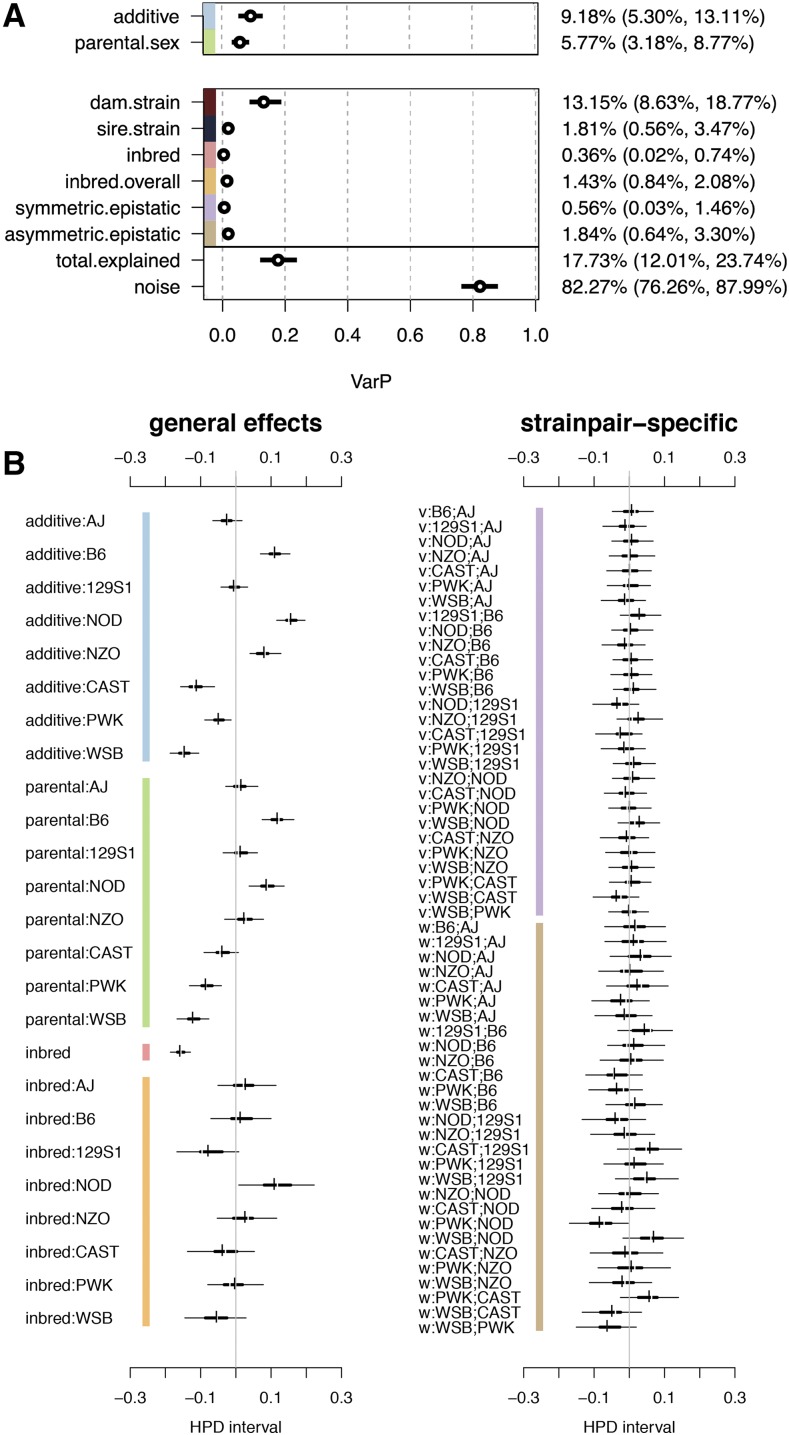
Diallel effects and variance contributions for weaned litter size. (A) Variance contributions of distinct effect classes, reported as posterior means and 95% HPDs of variance projections (VarPs) on weaned litter size. (B) Diallel effects, including (left) strain-specific additive, parental sex, and inbred effects, and (right) epistatic effects between each pairwise cross. For each parameter, thin and thick horizontal lines represent 95% and 50% highest posterior density (HPD) intervals of effects, respectively, and vertical break and dash give posterior median and mean, respectively. The effects are in relation to an overall mean litter size of 5.46 (95% HPD: 5.00-6.10). The gray vertical lines indicate zero. Effects are shown as the log, or latent, scale effects on the mean litter size attributable to each strain or strainpair and inheritance group, where values are centered at 0 for each random effect class. Intervals that exclude zero have non-negligible effects on the mean litter size. Labels with “v” or “w” refer to symmetric or asymmetric epistatic effects, respectively. Colored bars indicate corresponding variance classes in (A) and (B).

To estimate founder strain effects on litter size, we used a Bayesian regression model that decomposes the phenotypic variation in the diallel into genetic and parent-of-origin contributions (Lenarcic *et al.* 2012). Using this model, the percentage of the variance in litter size explained by diallel effects was 17.73%, with additive effects explaining 9.18% (VarP[additive]; this GCA-like measure being related to narrow sense heritability), parent-of-origin effects (VarP[parental.sex]) accounting for 5.77%, the fact of being inbred (VarP[inbred.overall]) at 1.43%, and strain-by-strain interactions (VarP[epistatic.symmetric] + VarP[asymmetric.epistatic]) at 3.40% (Figure 2A).

In more detail, we present estimates for all modeled diallel effects as posterior means and highest posterior density (HPD) intervals (Figure 2B). Parameters are divided into two groups: general effects and strain pair-specific effects. General effects comprise strain-specific additive effects (additive), strain-specific and overall inbred (inbred), and strain-specific parent-of-origin (parental sex) effects. Strain pair-specific (epistatic) effects are the effects that arise specifically in crosses of two heterologous strains, with ‘v’ referring to symmetric epistatic and ‘w’ referring to asymmetric epistatic effects (pairwise parent-of-origin effects).

Under the general effects, we see significant positive additive effects on average litter size from B6, NOD, and NZO and significant negative additive effects on litter size from CAST, PWK, and WSB strain dosages. A similar pattern is seen in the parental sex effects, where B6 and NOD dosages have a significant positive effect on average litter size, whereas CAST, PWK, and WSB have significant negative effects. The overall “inbred” effect is negative, indicating that inbred status decreases average litter size, regardless of parental strain. Each strain also has an individual inbred effect in addition to the overall inbred effect. When the individual inbred effects are taken into account with the overall “inbred” effect, inbred litters are on average slightly smaller than their heterozygous counterparts. For strain pair-specific epistatic effects, there are a few marginally noteworthy effects, with the most prominent being a negative asymmetric epistatic effect for PWK×NOD.

**Figure 3:**
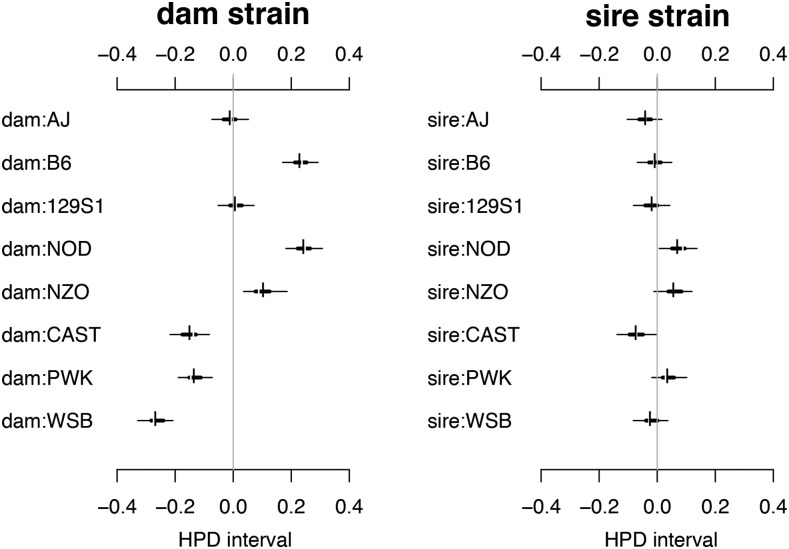
Dam.strain and sire.strain variance contributions and estimates of effects on weaned litter size. These effects are a reparameterization of additive and parental.sex effects from the previous analysis. Estimates for the maternal (“dam.strain”) and paternal (“sire.strain”) effects on litter size, as calculated from the additive and parental sex parameters in Figure 2, with HPD intervals defined correspondingly.

To more clearly differentiate the contributions of the mother *vs.* the father strain, we reparameterized the BayesDiallel model to capture effects specific to dam.strain and sire.strain (Figure 3). B6 and NOD dams increase litter sizes by more than 1.26 fold, by an average of 1.66 and 1.79 pups, respectively, regardless of sire. CAST, PWK, and WSB dams tend to decrease average litter size by 0.90, 0.81, and 1.51 pups. The sire effect is similar, with NOD and NZO sires having larger litters and CAST sires producing smaller litters. As expected, we see that the dam.strain has a much larger influence on the variation of litter size compared with the sire.strain (13.15% *vs.* 1.81%).

### No significant strain effects on sex ratio

We examined genetic and non-genetic effects on the average sex ratio per litter and found no evidence that sex ratio was skewed (Figure S6; Figure S7). The overall mean for sex bias, quantified as number of male pups weaned divided by the total number of weaned pups, was 0.4979, and did not significantly differ from our expectation of a 50:50 sex ratio (binominal test, two-tailed *P* = 0.219). For the eight inbred founders, 129S1 is the only strain that departed significantly from expectation, with slight reduction of males 0.475:0.525 (binominal test, two-tailed *P* = 0.041). However, correction for multiple testing shows no significant sex ratio bias of any inbred strain. The outbred crosses have substantially fewer litters and offspring than the inbred matings, leading to less balanced sex ratios; however, when multiple testing is accounted for, they also show no significant deviations from expected sex ratios.

## Discussion

We have investigated factors influencing litter size in the eight CC founders and their F1 hybrids using a new extension of the BayesDiallel model. We note that litter size is a component of reproductive performance, but distinct from total strain productivity; a study on total strain productivity would need to take into account litter size, numbers of litters, maximum reproductive age, and pup survival until breeding. Our results illustrate how mammalian litter size in a full diallel design is influenced by genotypic and environmental variation. These results present new information on CC founder strains’ reproductive performance, show that maternal effects and the environment play a large role in litter size variation, and provide no evidence for seasonality effects on litter size in a controlled animal facility. The results also address some of the factors that contributed to line extinction and breeding problems in generating the CC (Chesler *et al.* 2008; Philip *et al.* 2011; Collaborative Cross Consortium 2012; Shorter *et al.* 2017).

We estimated that genetic (additive, inbred, and epistatic) effects on average litter size explained 17.73% of variation, suggesting that most of the phenotypic variation arises from unexplained environmental effects. Compared with the overall average litter, we observed substantial positive effects of B6 and NOD strains, from both additive genetic and parent-of-origin parameters, and substantial negative effects of PWK and WSB (Figure 2). We also observed, as expected, that lower litter size was associated with being inbred (Figure 2A). These estimates are likely driven by the unique selection history of these inbred lines, and comparisons should be limited to these eight founder strains, and the CC and DO populations.

During the G1 and G2 out-crossing generations of the CC, mean litter size was lower for crosses involving wild-derived strains, CAST, PWK and WSB (Philip *et al.* 2011). A similar pattern is observed here, but these effects are determined to be specifically through the maternal strain, with CAST, PWK and WSB having negative “dam” effects on litter size (Figure 3). It is likely that selection pressure in classical lab strains is associated with larger litters compared with the wild-derived strains. Additionally, two of the wild-derived strains, CAST and PWK, are from a different subspecific origin than the other six CC founders. This likely contributes to decreased productivity through subspecific incompatibilities (Shorter *et al.* 2017).

We identify and report environmental factors that may influence litter size. The breeding of this diallel was performed across two different vivariums over the course of 4 years, and we see a significant effect from housing facility. The Hillsborough facility was associated with larger litters for all strains, especially 129S1 and WSB. The two facilities have many different factors that could explain these differences. The Hillsborough facility housed multiple species, including dogs and mice, had smaller rooms that held approximately 300 mouse cages, was remotely located in a rural area, had different laboratory personnel, had cage changes once a week, and was supplied with filtered well water. The diallel breeding at the GMB facility took place in a large central room, contained only laboratory mice, is located in a basement of a large seven story research building on campus, has cage changes every other week, and is supplied with filtered city water. It is possible that one or more of these factors, independently or in combination, affected productivity. Another finding was that seasonality, which has previously been shown to influence litter size and frequency of litters in mammals (Drickamer 1990), did not seem to significantly impact litter size in this study. This is likely due to consistent light-dark cycles and temperatures as well as a steady diet. We did observe a significant effect on litter size after the transfer of the mice to the UNC GMB facility (February 2010), which reduced overall litter sizes from March to June 2010. This may have been due to the use of Fenbendazole during the time of the transfer. Other factors, such as the sex of the laboratory personnel interacting with the animals, are generally known to influence rodent behavior and could contribute to some of the environmentally-induced variation we observed (Sorge *et al.* 2014).

Last, we measured sex ratio across all inbred and outbred crosses. Despite some departures from equality at the nominal significance threshold (alpha = 0.05), no associations with founder strain dosage remain significant after correction for multiple testing. Recent work has suggested a potential for bias in sex ratio driven by the male germline in *Mus musculus* (Conway *et al.* 1994; Macholán *et al.* 2008; Cocquet *et al.* 2009; Ellis *et al.* 2011; Cocquet *et al.* 2012; Turner *et al.* 2012; Larson *et al.* 2016), particularly in inter-subspecific hybrids that are mismatched for copy number of X- and Y-linked genes expressed in postmeiotic spermatids. Although there are no previous observations that suggest a bias in sex ratio in the CC or its founders, it remains an important characteristic to measure in a study on reproductive productivity.

To estimate heritable effects on liter size and sex ratio we extended the original BayesDiallel model of Lenarcic *et al.* (2012) in two new ways. First, to better understand and distinguish the effects arising from female and male parents, we reparameterized our strain-specific additive and parental-sex effects such that we could provide estimates of maternal strain and paternal strain effects separately. In the original BayesDiallel model, maternal and paternal strain effects are split into “additive” effects, which consolidates the effects they have in common, and “parental sex”, which models any remaining deviation between the two. Recognizing that additive effects from the sire are essentially wiped out by the additive effects, we instead collapsed the additive and parental-sex effects into “dam.strain” and “sire.strain” effects in a post-processing step on the posterior output. This allowed us to run the original BayesDiallel model while also viewing our data from the perspective of dam strain and sire strain contributions.

Second, we reimplemented the original MCMC sampler, designed for modeling a continuous outcome variable, in a general package MCMCglmm (Hadfield 2010) in order to model count and binary responses. Litter size, as measured, is most naturally distributed as Poisson, with zero-truncation owing to the fact that only successful litters were recorded. Sex ratio is most naturally modeled as a binomial, with an underlying (male) proportion between 0 and 1. Although it would be possible to obtain an approximate analysis by transformations to normality using the original BayesDiallel (and we do this for litter size for some otherwise hard-to-obtain quantities), we found such approximations to be inadequate for reliable estimation of higher order effects in the case of litter size and deeply flawed in the case of sex ratio. Although there is a computational cost, and added complexity to determining variance contributions, this new implementation achieves several objectives: 1) we no longer break the assumptions in the original model regarding normally distributed errors; 2) we easily accommodate overdispersion in our data; and 3) we can select from a large number of GLMMs models that more closely resemble the forms of our data observations. In addition, we believe this flexibility will be appealing to many other researchers who would like to model non-Gaussian distributed phenotypes using diallel designs, and we have provided the code in an R package litterDiallel (https://doi.org/10.5281/zenodo.2580307).

Overall, these results have implications for other avenues of future research. Future multiparental research populations should test for strain incompatibilities, reproductive phenotyping, and other health traits in a full diallel before the recombinant inbreeding begins (Odet *et al.* 2015). These future research populations should also use non-related wild-derived individuals from the same subspecific origin in order to increase genetic diversity without introducing hybrid incompatibilities.

## Figures and Tables

**Figure 4:**
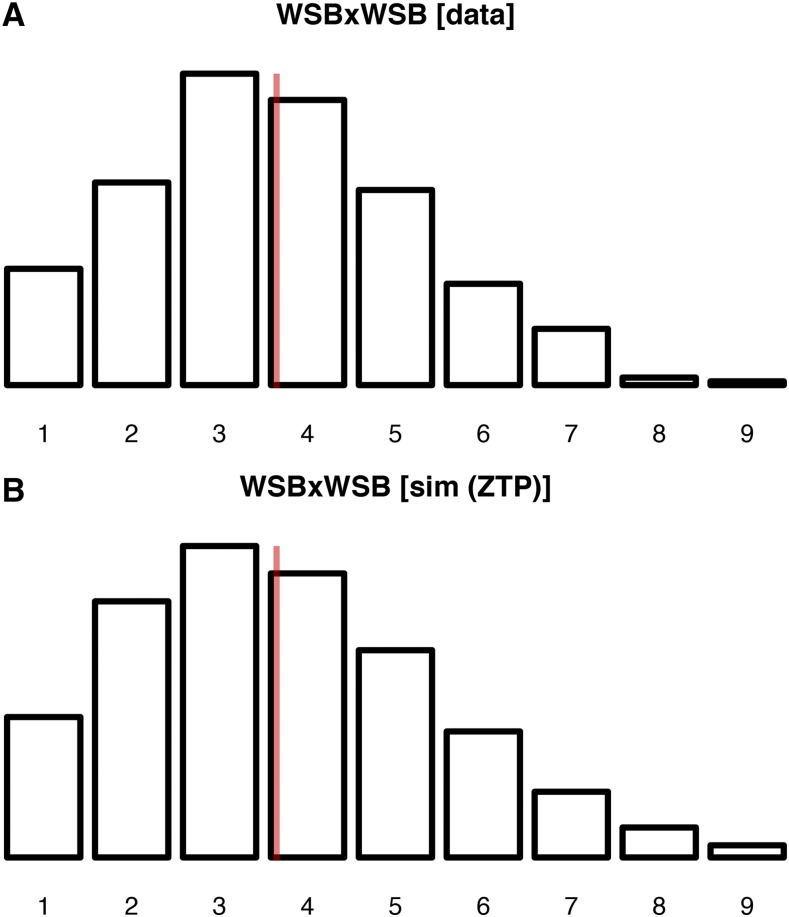
Comparison of observed data and (pseudo-)randomly generated data for the WSB×WSB inbreds. (A) Distribution of the litter diallel data for WSB×WSB (nlitters = 341, npups = 1,244). (B) Frequencies expected from a zero-truncated Poisson distribution with mean=3.648 (or λ=3.542, same as in A). The vertical red lines indicate the mean values.

**Figure 5:**
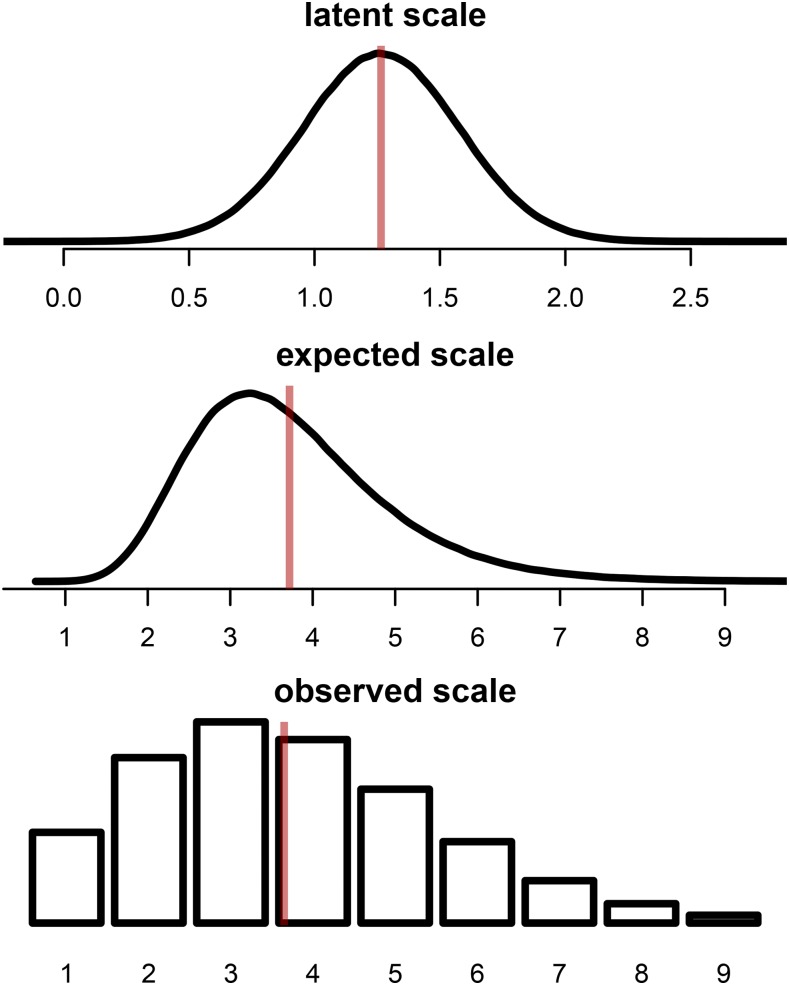
Latent and data scale comparison. An example illustrating zero-truncated Poisson regression of count data, with overdispersion, giving values drawn from pseudorandom normal variables drawn from N(mean=1.265,sd=0.311), showing how the continuous *latent* scale values [top], which correspond to the scale of the linear predictor, map onto their *expected* (data) scale value (through the inverse link, $f\left(x\right)=e^{x}$) [middle], and how the mean *expected* (data) scale value corresponds to the *observed* (data) scale values (integers) from ZTPois(λ=3.542) [bottom]. These values were chosen to resemble the real distribution shown in Figure 4. The vertical red lines correspond to the mean of the values shown in each respective frame.

## APPENDIX A

### Diallel Model For Litter Size

We collected data on litter size at weaning for 62 genetic crosses of inbred lines, across four years of breeding.

We use zero-truncated poisson (ZTP) regression for modeling our data. This type of regression is explicit in its framework accounting for discrete observations, flexible in its ability to use linear mixed models on the latent scale, and allows for parameterization of excess variance observed, in a way that standard Poisson regression does not. We account for the zero depletion in our data by using ZTP regression instead of standard Poisson regression, since we exclude observations of birth cohorts where no pups survived to weaning.

In Figure 4, the distribution of the observed data (litter size) is displayed for the WSB×WSB inbred mating, along with simulated data from a zero-truncated Poisson distribution based on the data mean.

For the ZTP, the first two moments (mean and variance), for values $y_{i}>0$, are given by:

$$E\left[y_{i}\right]=\frac{\lambda_{i}}{1-e^{-\lambda_{i}}}$$ (5)

$$V\left[y_{i}\right]=\frac{\lambda_{i}}{1-e^{-\lambda_{i}}}\left(1-\frac{\lambda_{i}e^{-\lambda_{i}}}{1-e^{-\lambda_{i}}}\right)$$ (6)

The density for the ZTP, for every count x∈1,2,..., is given by:

$$\text{ZTPois}\left(x|\lambda \right)=\frac{\text{Pois}\left(x|\lambda \right)}{1-\text{Pois}\left(0|\lambda \right)}$$ (7)

The relationship between the latent, expected, and data scales of the ZTP regression model are illustrated by the toy example shown in Figure 5.

ZTP frequencies were calculated using the R package countreg (Zeileis and Kleiber 2018).

### Diallel effect estimates

Diallel effect estimates are obtained using an MCMCglmm (Hadfield 2010) implementation of BayesDiallel (BayesDiallel-glmm, henceforth), with $1.515\times 10^{6}$ iterations, $1.5\times 10^{4}$ iterations of burn-in, and thinning by 500 (saving only every 1/500^th^ iteration), to obtain 3000 independent samples with minimal autocorrelation, and plotted as highest posterior densities (HPDs) in the results.

### Conversion to the expected data scale

The effects estimated from the BayesDiallel-glmm model are transformed from the latent scale to the (expected) data scale via the inverse link function, *i.e. $\exp\left(a_{AJ}\right)$*, for interpretability of effects on the original data scale.

### Variance Explained Using Variance Projection

To avoid the problem of interpretability in transforming variance parameter (and variance projection) estimates from the latent to the observed data scales, we instead calculate and report variance projections, as calculated using the Gaussian version of BayesDiallel. In order to account for heteroscedasticity (unequal variance) of the model residuals that arises from the approximately ZTPoisson nature of the data, we use a variance-stabilizing transformation (VST) (Yu, 2009) and run BayesDiallel again (Gaussian) to obtain Variance Projections on the modeled data. The VarPs that are calculated from these parameter estimates are an approximation of the variance contributions that we would observe in the GLMM BayesDiallel model.

## APPENDIX B

### Diallel Model For Sex Ratio

We model the male pup counts and female pup counts jointly, using the BayesDiallel linear model, formulated for binomial GLMM regression. This model directly considers genetic effects on the imbalance in male *vs.* female pups by parameterizing the number of males and number of total pups (or the total, and the fraction of males in the total). The model is elaborated in the methods section of the main manuscript.

The proportion of weaned pups that are male, or equivalently, the proportion of weaned pups that are female, is approximated by a binomial distribution. In our data, the mean and the variance of male ratio are 0.494 and 0.063, respectively.

To generate the upper and lower 95% boundaries, as shown in Figure S6, for the expected phenotype under the null hypothesis of male pup proportion = 0.5, we used the qbinom function in the stats package in R.

To generate the upper and lower 95% boundaries, as shown in Figure S6, for the expected phenotype under the null hypothesis of male pup proportion = 0.5, we used the qbinom function in the stats package in R.
